# Mechanisms of anti-predator behavior in the great ramshorn snail (*Planorbarius corneus*)

**DOI:** 10.1371/journal.pone.0313814

**Published:** 2024-12-06

**Authors:** Andrew Talk, Sajeevan Vanatheeva

**Affiliations:** School of Psychology, University of New England, Armidale, NSW, Australia; Tokai University, JAPAN

## Abstract

Like vertebrate animals, some invertebrates have been shown to exhibit fear- or anxiety-like behavior while in apparatus that allow choice between sheltered, darkened spaces and open, lit spaces. The behavioral mechanisms by which invertebrates accomplish this behavior, and whether those mechanisms are similar across species, has not been fully studied. Across three experiments, we investigated possible behaviors that Great Ramshorn snails (*Planorbarius corneus*) could use to accomplish fear behavior while in presence of the odor of a predatory fish. In experiment one, we used a light/dark preference box to demonstrate reduced preference for exposed and lit areas caused by the predator odor. In experiment two, we used an open field apparatus to demonstrate an increase in refuge-seeking (thigmotaxis, or time spent near a wall) in diffusely lit but not dark environments caused by predator odor. In the same experiment we found the snails were photokinetic (they moved faster in the light than in the dark) but we saw no effect of predator odor on photokinesis. In experiment three, we conducted a second open field study with a directional light source and found no evidence of phototaxis (movement direction with respect to light), and no effect of predator odor on phototaxis. Thus, in our studies we found evidence for refuge-seeking as a mechanism for fear-like behavior in the presence of predator odor and little evidence for perhaps more computationally simple strategies of increased photokinesis and phototaxis.

## Introduction

The capacity for fear- and anxiety-like behavioral patterns has been found to occur across vertebrate and invertebrate species and appears to be ancient [1–4]. This may in part be due to the importance of the state during avoidance of predators and other environmental dangers [5–7]. Implications of this research include animal welfare concerns [8]. However, there are open questions about the information-processing substrates of emotion-like behavior in invertebrates and the extent to which they are similar across species.

### Tests of invertebrate anxiety-like behaviors

Anxiety-like states in rodents can be assessed in the laboratory by measuring a variety of types of behaviors, including social interactions, exploration of a light-dark box, open field exploration, elevated plus-maze exploration, shock-probe burying, and conditioned aversive contextual responses including fear-potentiated startle responses within a conditioned context [9] [10, 11]. Many tasks used to assess anxiety-related behaviors necessarily allow observations of behavior in the presence and absence of anxiogenic stimuli [6, 12]. Nocturnal animals such as rodents tend to show a preference for dark enclosed spaces, and assessing anxiety often involves measures of unconditioned approach and avoidance behaviors within apparatus that contain such spaces in addition to exposed, open, or well-lit spaces [13–15].

Scientists have argued for the existence of fear- or anxiety-like behavioral conditions in invertebrates at least since the initial demonstrations of aversive conditioning in Aplysia [16]. In particular, demonstrations of aversive context conditioning in marine gastropod mollusks provided examples of behaviors that are considered anxiety-like when displayed by rodents or humans. For example, development of potentiated startle responses within a context paired with shock have been argued to be anxiety-like [17]. Similar context-dependent startle was demonstrated in Aplysia after a context was paired with shock. The siphon withdrawal reflex was subsequently potentiated within the paired, but not in an unpaired context [18]. In another study, Hermissenda were exposed to aversive context conditioning involving high-speed orbital rotation within either the lit or darkened side of a light-dark box. The subjects later avoided the lit side if that side was paired, but no such light avoidance occurred if the dark side was paired or if they were not treated with rotation [19]. Further studies have shown that stressful stimuli modulate memory formation in molluscs similarly to the effect of stress on rodents and humans. When pond snails (Lymnaea stagnalis) are operantly conditioned in the presence of predator odor or other stressors, memory formation is enhanced or suppressed in a dose-dependent manner similarly to mammals [20, 21].

The open and enclosed areas of laboratory apparatus commonly used in studies of emotions of rodents have more recently been adapted to study behavioral patterns indicative of anxiety-like states within invertebrate species. These have included studies of crayfish behavior (Procambarus clarkii) in a plus-maze [2], fruit fly (Drosophila melanogaster) behavior in an open field [3], and the behavor of planaria flatworms (Dugesia dorotocephala) in a light-dark box [4]. Administration of anxiolytic drugs, including those that modulate of γ-aminobutyric acid type a (GABAa) or serotoninergic receptors, change anxiety-related behavior in various pancrustacea species, including crayfish, fruit flies, and amphipods [2, 3, 7]. Studies of anxiolytic drugs in mollusks however typically have used doses relevant to environmental contamination and have not typically found effects on anxiety-like behavior [22, 23]. In one study, aquatic snails were placed in a water tank that also contained a shelter under which they could hide. Addition to the tank of water taken from a tank that housed predatory fish caused the snails to reduce their activity and shelter. Administration of the common antidepressant and antianxiety drug sertraline had no effect on this behavior.

### Fear- and anxiety-like behaviors elicited by predator odor

Prey species exhibit diverse adaptations to evade predation, among them predator avoidance. Behavioral changes like decreased activity and seeking refuge upon detecting predator signals can lower the likelihood of predator-prey encounters [24]. In rodents, predator scents trigger stereotypical defensive and avoidance behaviors along with activation of the hypothalamic-pituitary-adrenal (HPA) axis, which regulates the body’s sympathetic stress reactions [25]. Innate defensive reactions triggered by predator odor offer a valuable and valid avenue for investigating behavioral, physiological, neural, and pharmacological aspects of fear or anxiety [26, 27]. (In mammals, fear and anxiety are overlapping behavioral states that have the same neurobiological basis. The difference between “fear” and “anxiety” is typically defined as relating to the duration of the eliciting stimulus [28]). Using ethologically pertinent anxiogenic stimuli such as predator odor in fact offers several benefits. These include the alignment of subject responses with their natural habitat and sidestepping confounding factors associated with pain reactivity.

Various aquatic snail species have been found to respond to predator odor with a variety of anti-predator behaviors. For example, the freshwater snail Physella gyrina responds to crayfish odor by moving to the surface and avoiding covered habitats but responds to predatory fish odor by moving under a cover provided on the bottom of the tank [29]. Pond snails have also been shown crawl above the water line when predator odor was added to the water, and this effect was amplified when combined with effluent from crushed conspecifics [30]. Pond snails also display faster righting responses in predator odor water if placed with their ventral part of the foot exposed and away from the substrate, but take a longer time to re-emerge from their shells after perturbation [31]. The pond snails also had a more reactive withdrawal response within predator odor water if presented with a passing shadow [31].

### Current studies

In the current studies we used a light-dark box and two versions of the open field test. A light-dark box is a box divided into a lit and open section and a darkened and enclosed section. When used with rodents, an anxiety-like behavioral state is inferred by measures of time spent in the lit, exposed section or sometimes by latency to enter the light [32, 33]. The open field apparatus is an open arena wherein an animal is allowed to freely explore for some amount of time. Anxiety-like states in rodents are inferred by locomotor activity, reported most often as speed or amount of movement over time. Moreover, time spent away from the walls of the apparatus and in the center are also measures of anxiety-like behavior [33–35]. putative stress-inducing experiences such as restraint, shock, and predator odor decrease exploratory behavior, particularly of open and lit areas [27, 36].

It is not clear by which behavioral or cognitive mechanisms that the various invertebrate species possess the capacity for similar anxiety-like behaviors, or whether they are always similar to the mechanisms of vertebrates. For example, a randomly moving organism could effectively reduce time in lit areas simply by increasing movement speed in the light and slowing in the dark, or an organism could avoid light by actively turning and moving away from the source of the light [37]. We thus conducted the present set of studies to assess these potential aspects of behaviors induced by predator odor in a gastropod mollusk species. We first sought to demonstrate reduced preference for exposed and lit areas induced by predator odor in a light/dark preference box. We made observations across the transition light to dark phase of the circadian rhythm so we could observe the snails at times in which there was an illumination level difference and also when both sides of the box were equally dark. Then, in additional studies conducted in two sets of open field apparatus, we sought to determine the mechanisms by which this behavior occurs. We assessed the effect of predator odor on photokinesis (speed in a lit versus dark environment), thigmotaxis (time spent near a wall) in lit vs dark environments and phototaxis (movement direction with respect to a light source).

## Methods

### Husbandry and housing

Great ramshorn snails (*Planorbarius corneus*) occur in still or slowly moving waters of Europe, where they can occur at population densities of up to 30 individuals per m^2^ [38]. Natural predators include local molluscivorous fish such as perch or trout. However, they are commonly sold as aquarium pets and have been introduced into the wild worldwide [39]. The source of the original snails for our colony was an aquarium shop.

We laboratory bred and raised great ramshorn snails in 50 L tanks of artificial pond water made with aged tap water and commercial aquarium electrolyte supplements (Seachem and Marine Master). Pieces of dead and sterilized coral were placed in the tanks to supplement calcium concentrations. The tanks were fitted with aquarium lights and were maintained at 21 ± 1°C on a 12 h: 12 h light: dark cycle (lights on at 7 am). The snails were fed iceberg lettuce (*Lactuca sativa*) ad libitum and had half of their tank water replaced weekly. The snails were used as subjects when they were 10–12 weeks old. At the conclusion of each study, we euthanized the snails we used by freezing. New cohorts were used for each study.

### Apparatus and stimuli

The experiments were conducted using either control water or predator odor water. To prepare these, we pureed and strained 300 grams of iceberg lettuce in 1.25 liters of aged tap water, then diluted it to achieve a concentration of 10 grams of lettuce per liter in the experimental chambers. The control water was simply the water with added shredded and strained lettuce. For the predator odor water, we additionally pureed and strained 5 grams of barramundi (Lates calcarifer) skin in 250 milliliters of aged tap water. This mixture was then added to the control water, resulting in a final concentration of 20 milligrams of pureed barramundi skin per liter along with 10 grams of lettuce per liter. Barramundi are opportunistic predatory fish that consume mollusks, arthropods, and fish [40]. They are commonly farmed for food, and the skin used in the study was sourced from a local grocery store.

The light/dark boxes consisted of four rectangular (600 l x 60 w x 240 h mm) glass tanks. We wrapped half of each tank with aluminum foil so that the top, sides, and bottom of half of the tank was enclosed and protected from light. We placed the tanks side by side on a glass tabletop in a darkroom and filled each tank with 6 L of either control or predator odor water. We positioned a 55 cm long LED aquarium light 33 cm above the water surface across the center of the tanks so that it illuminated the open half of each tank. The illumination levels at the water surface were 280 lux on the lit side and 3 lux on the shadowed side. With the aquarium light turned off, the illumination levels were less than .005 lux on both sides of the tank. We used a Habotest HT620 for all light measurements.

The diffuse-light open field apparatus consisted of 14 cm square opaque plastic dishes that were filled to a depth of 1 cm with either control or predator odor water. Thirty-six such chambers were arranged on the floor in a darkroom. Four 35 W fluorescent lamps were situated to the sides of the dishes providing indirect room illumination. Illumination at the water surface level was 180 lux with the lamps on and less than .005 lux with the lamps off. The snails were imaged from above using an infrared USB camera.

The directional-light open field apparatus were six flat-bottomed round clear Pyrex dishes that were placed on a glass tabletop in a darkroom and filled to a depth of 2 cm with either control or predator odor water. The dishes were each set on the top and center of white paper that had a circle printed on it with a radius of 7 cm. A 3 W LED lamp was diffused through opaque white plastic to provide a light source to the side of the dishes 30 cm from the dish centers. Illumination was 60 lux at the water surface on the sides of the dishes nearest the lamp and 20 lux on the sides of the dishes furthest from the lamp. The snails were imaged from above using an infrared USB camera.

### Procedures

#### Light/dark box test

We placed four light/dark box tanks side by side on a glass tabletop in a darkroom and filled two tanks with 6 L of control water and two with predator odor water. At 3 pm, we placed 40 snails at the center of each of the four tanks. The snails were allowed to freely explore for the subsequent 16 hours. The snails were directly observed at 5, 6, 7, and 10 pm and then at 7 am the following day. The number of snails in the open half of each tank was noted. Snails were scored as being in the open side only if their entire foot was on that side. The aquarium lamp was turned off immediately after the 7 pm observation so subsequently both the open and enclosed halves of the tanks were in the dark. For the 10 pm observation, we briefly illuminated the tanks using the darkroom safelight only for the amount of time needed to make the observations. The overhead lights were turned on at 7 am and we immediately made the 7 am observations (before the snails had time to move from the positions they held in the dark). We then removed the snails to holding tanks. The light/dark tanks were emptied, cleaned with ethyl alcohol, and prepared for the second day of the experiment.

We ran the experiment identically on the following day, with snails that received the control water on the first day receiving the predator odor on the second day, and snails that received the predator odor on the first day receiving the control water on the second day. After the 7 am observation the following day, the lights were left on, and the snails were returned to their respective home tanks. At 9 AM, each snail was checked for viability (that they were upright and clinging to the substrate).

#### Diffuse light open field test

We conducted the study between 1 and 6 PM on a single day with a new cohort of snails. We arranged 36 open field chambers on the floor in a darkroom and filled 18 of them with predator-odor water and 18 with control water. We placed naive snails Individually in the center of each of the open fields and held them until they became attached to the substrate. They were then allowed to move freely throughout the apparatus. After a 10-minute acclimation period, the fluorescent lamps were turned on or off every 15 minutes for 1.5 hours. Following the open field exposure period, we rinsed each of the snails in control water and placed them individually into separate holding chambers.

A new set of open field apparatus were used for the study replication. The replication was run identically to the first study except that each snail was placed into an open field containing the water type they had not yet experienced. Snails that first received control water were placed into predator odor water, and snails that had received predator odor water were placed into control water. After a 10-minute acclimation period, each subject was then allowed to move freely about the open field for a further 1.5 hours with the fluorescent lamps turning on or off every 15 minutes. Each snail was then checked for viability (that they were upright and clinging to the substrate) before removing them to a holding tank. The coordinates of each snail were determined at each 30 second time point using ImageJ Manual Tracking plug-in. The observer doing this was blind to the independent variable of water type. Two dependent measures were collected: Photokinesis was indexed as movement speed in the light vs the dark. These data were calculated by determining the distance moved across each 30-second sample point and dividing by the 0.5 min elapsed time. Thigmotaxis was indexed as percent time spent within .8 cm of an apparatus wall.

#### Directional light open field test

We conducted the study between 3 and 6 PM across two days. We arranged 6 open field chambers on a glass tabletop in a darkroom and filled 3 of them with predator-odor water and 3 with control water. We placed snails individually in the center of the dishes at random orientation with respect to the light source and held them in place until they became attached to the substrate. They were then allowed to move freely about the dish until they crossed the 7 cm radius circle. They were then returned to a holding tank. We ran each subject only once. Phototaxis was indexed by determining the location of the snails as they crossed the 7 cm circle in ImageJ and then calculating the angle to the light source location with respect to the location of the center of the dish using Microsoft Excel. Thus, if the crossing point was directly between the dish center and the light the angle was 0° and if directly away the angle was 180°. We also recorded the time it took for each snail to cross the circle from the center. One animal was found to be not viable at the end of the study, and the data from that animal was not used.

### Statistical analysis

We performed statistical analyses using the software SPSS. For Experiment 1, we compared number of subjects in the open side of the light/dark test tank at each of the 10 time points. We conducted Chi-square tests for these observations due to their nonparametric nature. Even though this study has a repeated measures design, we did not track the subjects across observations. We thus took a conservative approach in the statistical analysis by treating each observation as independent. We applied a Bonferroni correction to the critical alpha level by dividing it by ten, the number of comparisons we tested. Photokinesis during the diffuse light open field Test was analyzed using a repeated measures ANOVA on the rate of movement with the variables of water type (control or predator odor water) and illumination level (light or dark). Thigmotaxis in the diffuse light open field test was analyzed using a repeated measures ANOVA on the percent time spent near a wall with the variables of water type (control or predator odor water) and illumination level (light or dark). Being near a wall was defined as the center of the snail as being less than 0.8 cm of the nearest wall. To assess phototaxis with reference to a light source in the directional light open field test, we calculated the Rayleigh statistic for the uniformity of a circular distribution [41] in Microsoft Excel. Also, we performed a MANCOVA with the angle and outward speed to the 7 cm circle serving as the dependent measures and control water or predator water odor as a between-group factor.

## Results

### Light-dark box test

**Fig 1:**
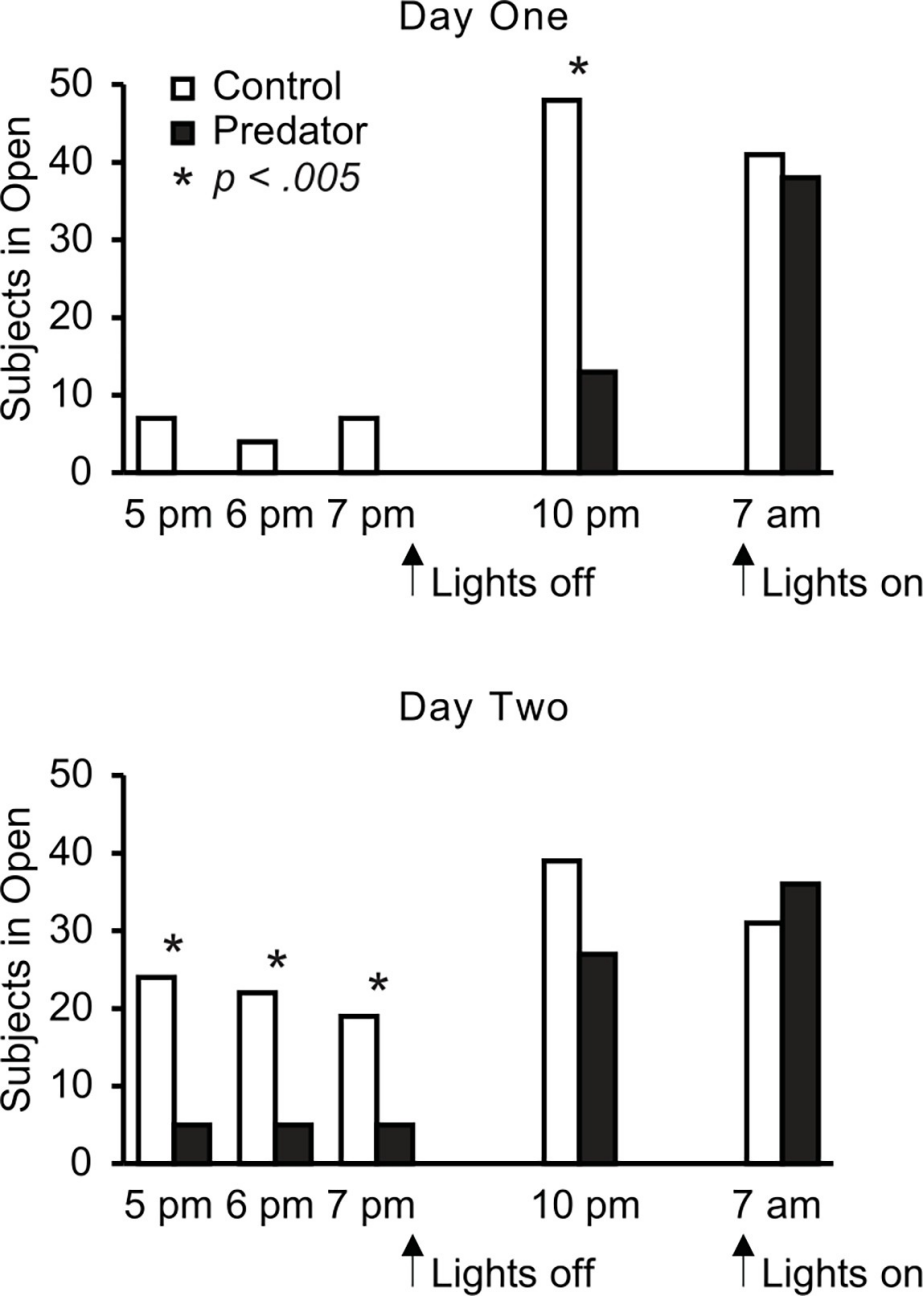
Number of subjects observed in the open side of the light-dark box apparatus at each observation time point. Subjects were in either control water (white bar) or predator odor water (black bar). The light turned off immediately after the 7 pm observation and immediately before the 7 am observation. Light levels in the open side were brighter than in the enclosed side with the light on. With the light turned off, on both sides of the tank were dark. Asterisks denote comparisons of p < 0.005. Note there is no black bar indicated for Day One 5 pm, 6 pm, or 7 pm because no subjects were observed in the open side of the tanks at those times.

More subjects of the control water condition than of the predator water condition were in the open chamber of the light–dark box At the 10 pm observation of Day One (*X*^*2*^ (1, N = 80) = 32.46, *p <* .*001*), and at the 5 pm (*X*^*2*^ (1, N = 80) = 15.20, *p <* .*001*), 6 pm (*X*^*2*^ (1, N = 80) = 12.88, *p <* .*001*), and 7 pm (*X*^*2*^ (1, N = 80) = 9.61, *p =* .*002*) observations of Day Two (Fig 1). Differences observed at 5 pm (*X*^*2*^ (1, N = 80) = 7.32, *p =* .*007*), 6 pm (*X*^*2*^ (1, N = 80) = 4.10, *p =* .*043*), and 7 pm (*X*^*2*^ (1, N = 80) = 7.32, *p =* .*007*) on Day One, and 10 pm on Day Two (*X*^*2*^ (1, N = 80) = 3.70, *p =* .*054*), were not deemed statistically reliable after correcting for the number of comparisons we made. Differences at the 7 am observation (in the morning at the end of the dark phase) were also not statistically reliable for Day One (*X*^*2*^ (1, N = 80) = 0.23, *p =* .*64*) or Day Two (*X*^*2*^ (1, N = 80) = 0.64, *p =* .*42*).

In addition, a habituation effect can also be observed in subjects which are exposed to the predator odor and control odor. More snails were observed in the light on day two than day one. McNemar’s test determined that there was a statistically significant difference in the proportion of observations of snails in the light from day one to day two, (*X*^*2*^ (1, N = 480) = 60.02, *p <* .*001*).

### Diffuse light open field test

#### Photokinesis

**Fig 2:**
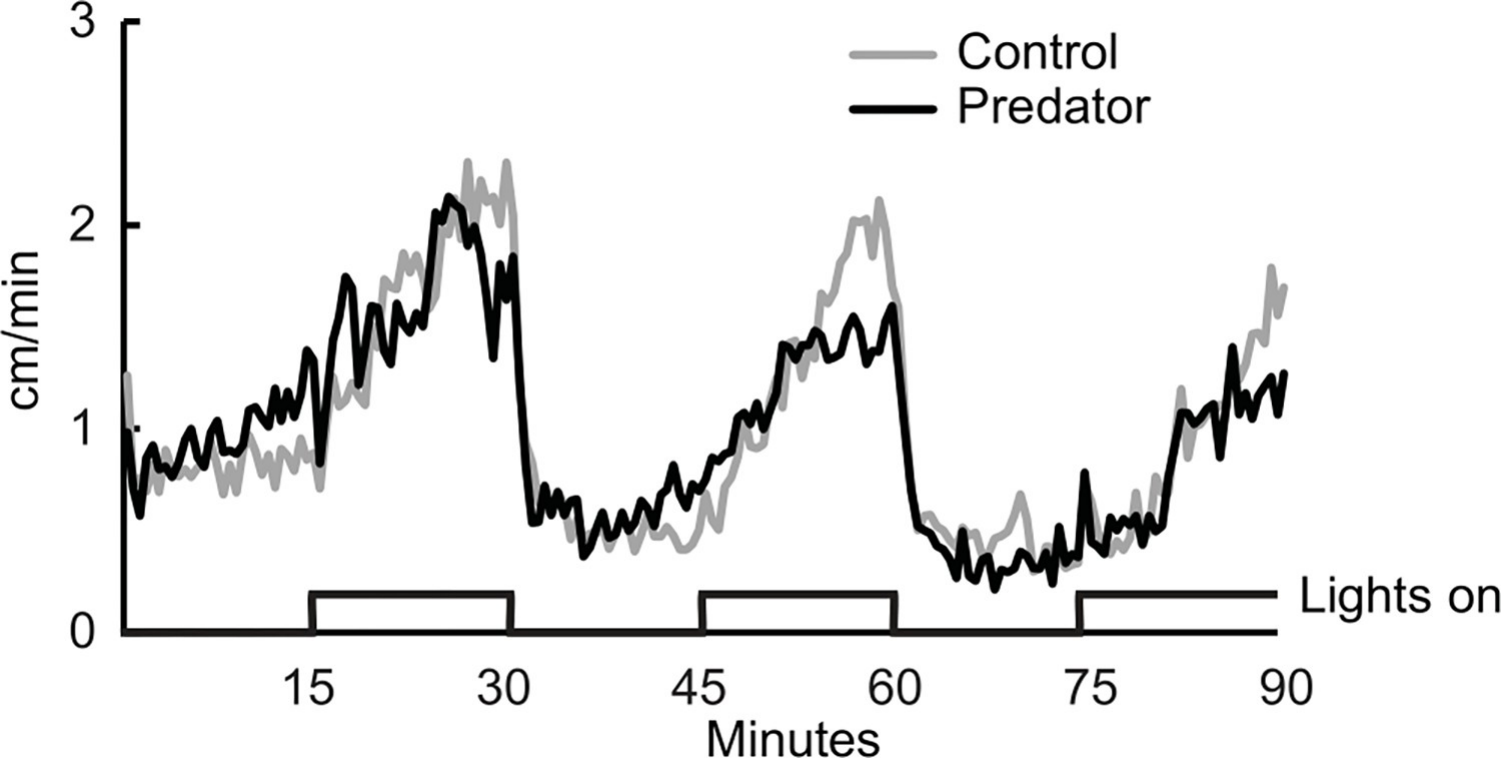
Observed speed of subjects for each 0.5 min time bin as they explored an open field apparatus in an alternately illuminated and darkened room. Lights on time periods are indicated at the bottom of the graph. Subjects were in either control water (grey line) or predator odor water (black line).

The snails moved faster in the light than in the dark (F_1,35_ = 46.22, p < .001; Fig 2). However, there was no reliable difference in rate of movement of snails while they were in the control odor water vs predator odor water (F_1,35_ = 0.02, p = .888). There was also no reliable interaction between water odor and light (F_1,35_ = 0.48, p = .495).

#### Thigmotaxis

**Fig 3:**
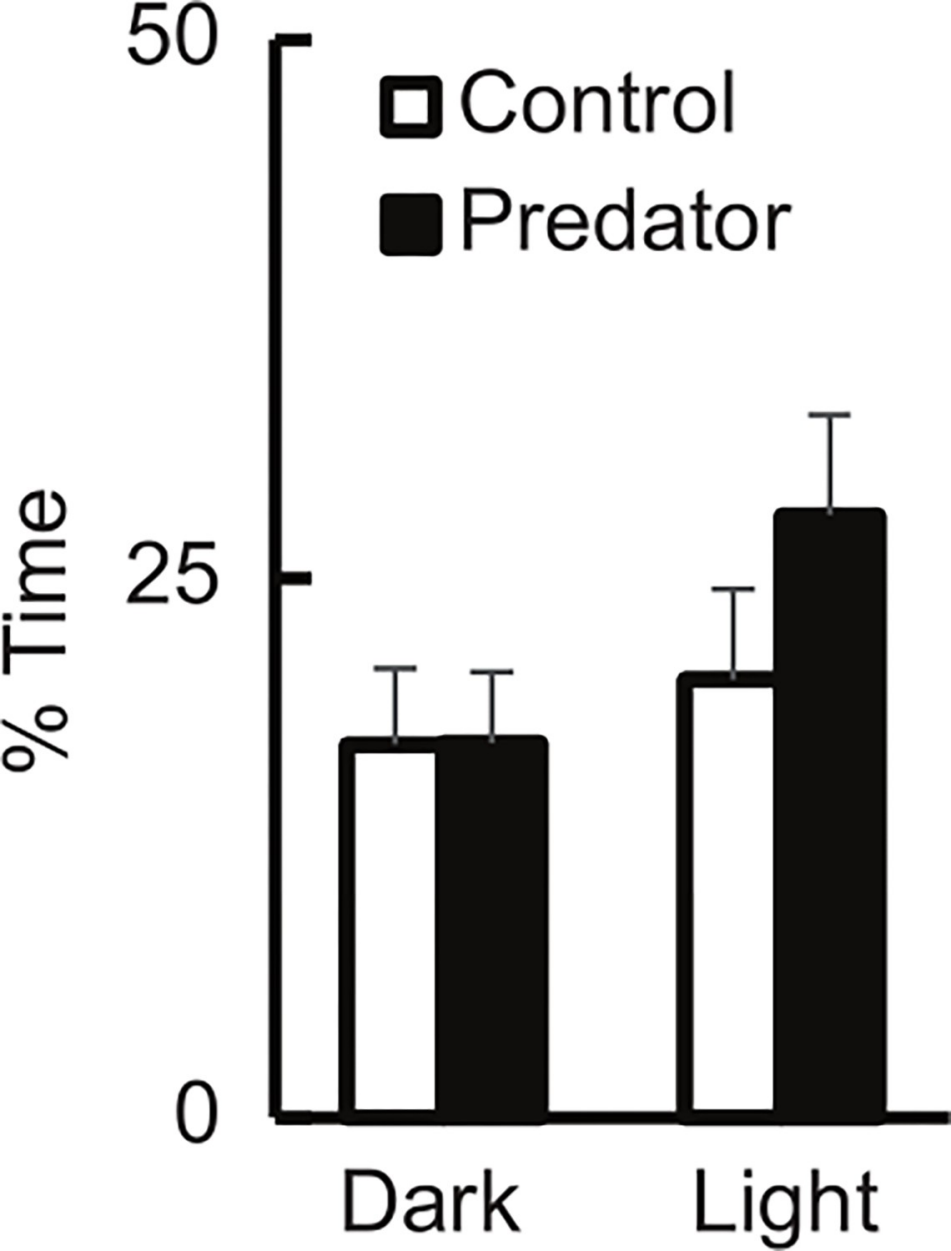
Percent time subjects spent adjacent to a wall as they explored an open field apparatus in an alternately illuminated and darkened room. Subjects were in either control water (white bar) or predator odor water (black bar). The error bars represent standard error of the mean.

There was a reliable interaction between water odorant type and lighting condition (F_1,35_ = 5.99, p = .019) so that the subjects spent more time within .8 cm of the wall in the light if in water with predator odor (Fig 3). The snails were also more likely to be in proximity to the wall in the light than in the dark (F_1,35_ = 16.05, p < .001). There was no reliable main effect of odorant type (F_1,35_ = .406, p = .528).

### Directional light open field test

#### Phototaxis

**Fig 4:**
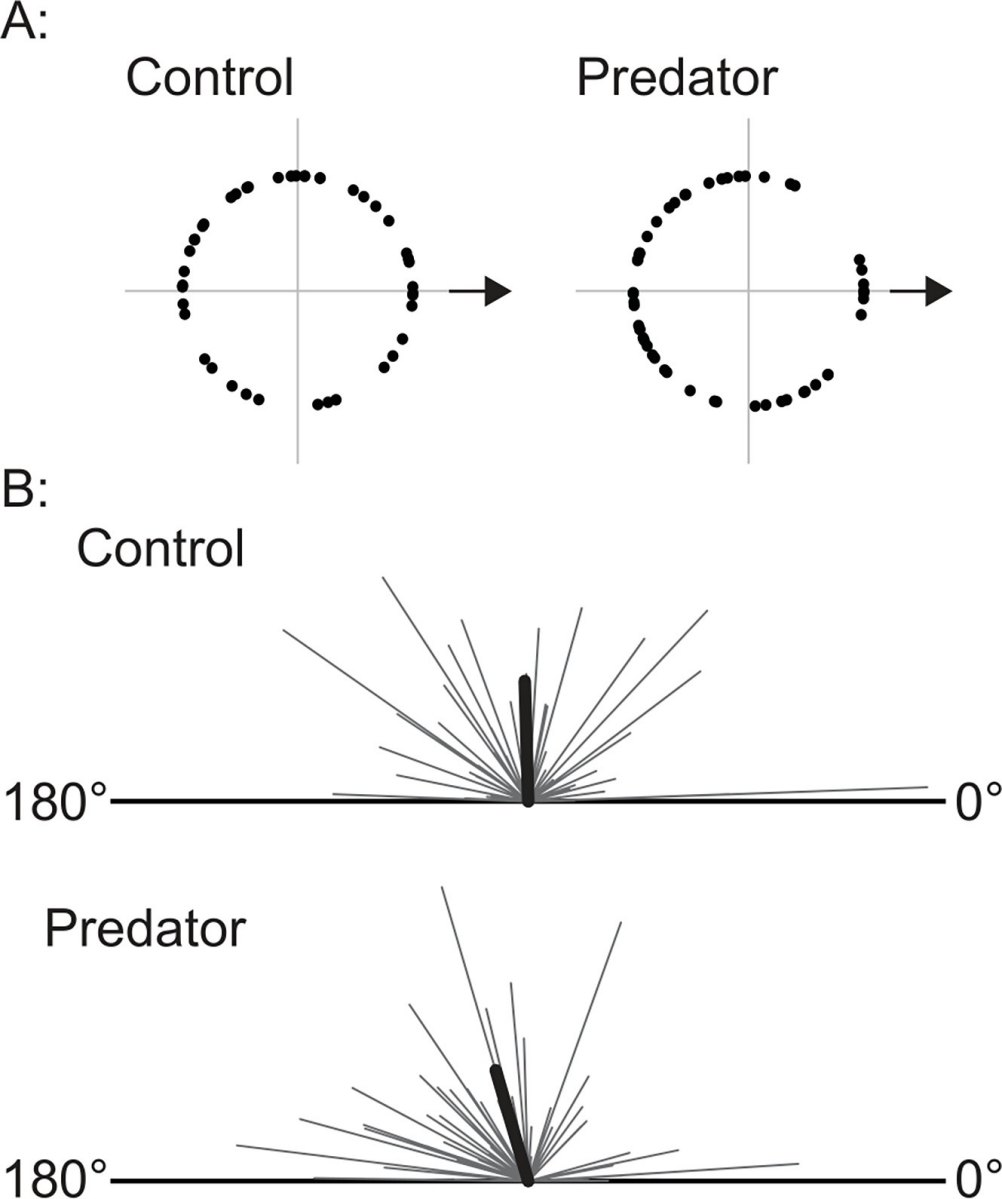
A: Location at which subject intersected the circle 7 cm from the center start location. The arrows indicate direction to the light source. B: Vectors for each subject that represent the average angle of movement with respect to a light source (0° = towards light source), and average outbound speed (length of vector). The average vector for each condition is represented by the thicker black lines.

Subjects that received the either the control or predator odor displayed no significant approach or avoidance behavior with respect to the direction a light source. Rayleigh’s Z-statistic of circular uniformity showed no reliable non-uniformity of movement direction for either subjects in the control group z = 1.54, or in the experimental group z = 2.77 (Fig 4). We also conducted a MANCOVA on dependent measures of angle and speed and between-group factor of water odorant (Control, n = 43; Predator, n = 52). It did not reveal a significant main effect for group F_(2, 92)_ = 0.843, p = 0.434. Subsequent univariate F tests revealed no significant main effect for group for the angle F_(1, 93)_ = 1.59, p = 0.21 or speed F_(1, 93)_ = 0.09, p = 0.76 scores. The mean angle and speed summary scores for the control and predator odor groups are shown in Fig 4 as vectors of angle towards or away from the light source and overall outward speed represented as the length of the vector.

## Discussion

Our initial goal with the current study was to assess the capacity of an aquatic gastropod mollusk to express preference or avoidance of exposed, illuminated areas of a light/dark preference box, and to determine whether this behavior is altered by having a predator odor stressor in the water. Then, using studies of behavior in an open field, we aimed to unravel some of the underlying information processing or behavioral mechanisms responsible for any observed behavior related to preference for dark, enclosed places or open, lit spaces with and without predator odor. Avoidance of lit or open spaces, particularly during or after stress, has been termed as “anxiety-like” when expressed by rodents (Reference with predator odor) or invertebrates [2, 7]. However, the extent to which the information processing or behavioral mechanisms underlying anxiety-like behavior is shared across species is open to exploration.

We found that Ramshorn snails reliably avoided the lit and exposed chamber of a light/dark box, and this avoidance was enhanced while in the presence of odor from a predatory fish. Then using an open field apparatus, we found that the snails expressed photokinesis, moving on average faster during periods of diffuse illumination. Moving faster in the light and more slowly in the dark could be one mechanism for avoidance of the lit areas in an environment [37]. However, we found no effect of predator odor, or light by predator odor interaction, on speed of movement. Photokinesis thus can’t explain the enhanced light avoidance in the presence of predator odor that we observed. We did find that snails expressed more thigmotaxic behavior, spending more time near the wall of an open field, when in illuminated rather than darkened surroundings. This particularly occurred within the presence of predator odor. These findings suggest that the snails may be using an anti-predator sheltering strategy, spending more time in protected locations while in the presence of predator odor in the light. However, note that thigmitaxis alone could not be an effective method of light avoidance because there were as many walls and corners in the light as in the dark. In a separate open field made of clear glass, we found that the snails did not express phototaxis. They instead moved randomly with respect to a light source at a particular direction. This behavior was also not reliably influenced by the presence of predator odor.

We conclude that the behavioral mechanisms of light avoidance in ramshorn snails are more likely to involve photokinesis rather than phototaxis. Anxiety-like behavior of the snails in an environment with both light and predator odor may additionally involve behavior related to sheltering. This was seen in our studies by snails remaining in protected areas including the darkened side of a light/dark preference test chamber, or near the wall of an open field apparatus during diffuse light. Further studies may be required to establish confidence about our null findings related to the influence of predator odor on photokinesis and phototaxis. For example, assessing effects of predator odor on photokinesis and phototaxis in a variety of lighting conditions would resolve any floor or ceiling effects we may have encountered.

With respect to comparisons with mammalian species, anxiety-like behavior in the open field in rodents and snails appears to be manifested with largely but not completely similar patterns of behavior. As we saw in snails, exposure of rats to increased light levels as opposed to dark increases thigmotaxis [42]. Exposure of rodents to predator odor inconsistently leads to anxiety-like behaviors, but as we saw here in snails, larger effects of predator odor in rodents occur within anxiety-provoking environments that also include bright lights [43]. One notable difference between our observations with snails and earlier studies of rodents in the open field is that in rodents, light decreases locomotor behavior [42] whereas we found that light increases movement speed. Thus, photokinesis might explain light-avoidance behavior in snails but it cannot explain light avoidance in rodents. Additionally, we documented habituation of avoidance of lit and open spaces in our light-dark box in a second test conducted at a 24-hour delay. Habituation also occurs with repeated testing of rodents in the light-dark box [32].

## Supporting information

S1 File (XLSX)
